# Lumbar osteopathic manipulative treatment can improve KOA symptoms: short-term efficacy observation and mechanism analysis

**DOI:** 10.3389/fbioe.2024.1431527

**Published:** 2024-08-22

**Authors:** Peiyu Du, Xi Li, Shilin Yin, Wenyi Li, Xilong Sun, Zekun Zhang, Jianyong Zhao, Gao Shijun, Shuangqing Du, Di Zhang

**Affiliations:** ^1^ Spine Surgery Department, The Third Hospital of Hebei Medical University, Shijiazhuang, Hebei, China; ^2^ Orthopedics Department, The First Hospital of Hebei Chinese Medicine University, Shijiazhuang, China; ^3^ Spine Surgery Department, Hebei Provincial People’s Hospital, Shijiazhuang, China; ^4^ Imaging Department, The First Hospital of Hebei Chinese Medicine University, Shijiazhuang, China; ^5^ Spine Surgery Department, Cangzhou Integrated Chinese and Western Medicine Hospital, Cangzhou, China; ^6^ Joint Surgery Department, The Third Hospital of Hebei Medical University, Shijiazhuang, China

**Keywords:** osteopathic manipulative treatment, manual therapy, knee osteoarthritis, mechanism analysis, spine-pelvis-lower limbs

## Abstract

**Background:**

Manipulative treatment can effectively improve knee pain and function, but no previous studies have shown that lumbar osteopathic manipulative treatment can improve knee symptoms. To explore the influence of lumbar manipulation on KOA and analyze its principlerelationship between coronal position of lumbar spine and KOA.

**Methods:**

Patients were divided into OMT group and DT group according to treatment. WOMAC scores were compared between the two groups, and X-ray examinations before and after treatment were performed in OMT group to analyze the imaging changes.

**Results:**

Both OMT group and DT group showed significant improvement in WOMAC score after treatment, and the improvement in OMT group was better than that in DT group. After OMT treatment, cTMI(*P* = 0.034), mL-SOD (*P* < 0.001), mΔL-KOD (*P* = 0.001), LL (*P* = 0.036), and FTA(*P* = 0.026) were significantly changed.

**Conclusion:**

Compared with drug therapy, lumbar manipulation can better improve WOMAC scores in KOA patients. It relives symptoms by loosening muscles and correcting small joint disorders to improve local knee alignment.

## Introduction

Knee osteoarthritis (KOA) is the most common degenerative disease in elderly patients, and it is also the disease with the highest teratogenic rate. The main clinical manifestations are pain and limited mobility (Horváth et al., 2011). At present, the first-line treatment of KOA is non-surgical treatment, which mainly includes physical therapy and drug therapy (Duong et al., 2023). Among them, the most commonly used non-steroidal anti-inflammatory drugs (NSAIDs) improve the pain symptoms of patients by inhibiting the sensitivity of PGs mediated chemical or mechanical receptors, and reduce the fear of exercise in patients, thereby improving the symptoms of limited activity in patients, but it will increase the risk of gastrointestinal complications and cardiovascular complications (Fallach et al., 2021), and there have also been studies that have shown poor efficacy (Adatia et al., 2012). In physical therapy, exercise therapy is gradually becoming the core therapy of KOA, but exercise therapy needs patients to adhere to long-term, its short-term effect on patients is poor, and patients can adhere to these exercises for a long time is not clear, and improper exercise will lead to further aggravation of the condition (Cinthuja et al., 2022).

Another kind of physical therapy, osteopathic manipulative treatment (OMT), its effect for KOA is still controversial. It has been confirmed in the literature that the symptoms and function of the knee pain of patients have been improved in the case of manual intervention of the knee joint (Reza et al., 2021), but there are also studies that suggest that the effect is not clear (Runge et al., 2022). However, manual treatment of the waist to improve knee symptoms has not been reported. In China, traditional Chinese medicine has a long history of treating KOAand pay attention to the concept of “Treat the upper to heal the lower” and “Holistic concept.” Based on this, traditional Chinese medicine has achieved good immediate results for patients with KOA through manual treatment of the pelvis and spine. In recent years, a growing number of studies have also linked knee pain to spinal and pelvic function (Pfluegler et al., 2021).

There has been no clear conclusion as to why OMT improves knee symptoms. Because most of the subtle changes caused by OMT and functional exercise cannot be directly measured by imaging examination, previous studies mainly evaluated the effect of OMT through indicators such as executive function score and range of motion (Currie et al., 2023). However, this does not provide an intuitive and rigorous explanation of the treatment mechanism, which is why many guidelines do not recommend manual therapy as a routine treatment for KOA (Paige et al., 2017). In this study, the standard standing posture was used to compare the full-length lower limb X-rays taken by the same patient in the standard standing posture before and after OMT, thus avoiding the error caused by the inconsistency of foot distance each time the patient stood naturally in the traditional full-length lower limb X-ray shooting, and then measuring the tiny parameters changed by OMT (Du et al., 2024). We hypothesized that lumbar OMT could improve knee symptoms, this study aims to explore how lumbar OMT can reduce the symptoms of KOA, at the same time, provides a new therapeutic idea for the conservative treatment of KOA.

## Methods

### Study cohort

This study was approved by the local ethics review committee and performed in accordance to the guidelines specified in the Declaration of Helsinki. Inclusion criteria: 1. Patients with KOA jointly diagnosed by two senior orthopedic surgeons. 2. Did not receive any treatment for KOA in the last 2 weeks. Exclusion criteria: 1. Patients with spinal coronal imbalance 2. Patients with severe osteoporosis 3. Suppurative infection, tumor, tuberculosis, trauma, etc., resulting in structural changes in the knee joint 4. Have serious heart, brain, kidney and other organic diseases, or mental illness episodes 5. Patients with previous knee surgery or lumbar surgery 6. Patients with incomplete clinical data.

This study was a retrospective multicenter cohort study. We collected 194 patients with KOA who were seen between 1 July 2023 and 31 December 2023. According to their treatment methods, they were included in OMT group and drug therapy (DT) group according to the inclusion and exclusion criteria. OMT Group underwent standard full-field lower limb radiography before and 2 weeks after treatment, and recorded the knee Western Ontario and McMaster Universities Arthritis Index (WOMAC) score. In the DT Group, WOMAC scores were recorded only before treatment and after 2 weeks of drug treatment. Define the side of the patient with more pain as the affected side and the other side as the healthy side. All patients voluntarily participated in this study and signed informed consent. Short-term efficacy was defined as 2 weeks after treatment.

### OMT group interventions

**FIGURE 1:**
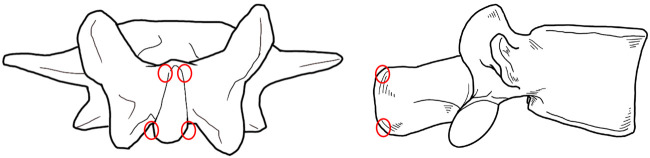
The press position of each spinous process (red circle).

**FIGURE 2:**
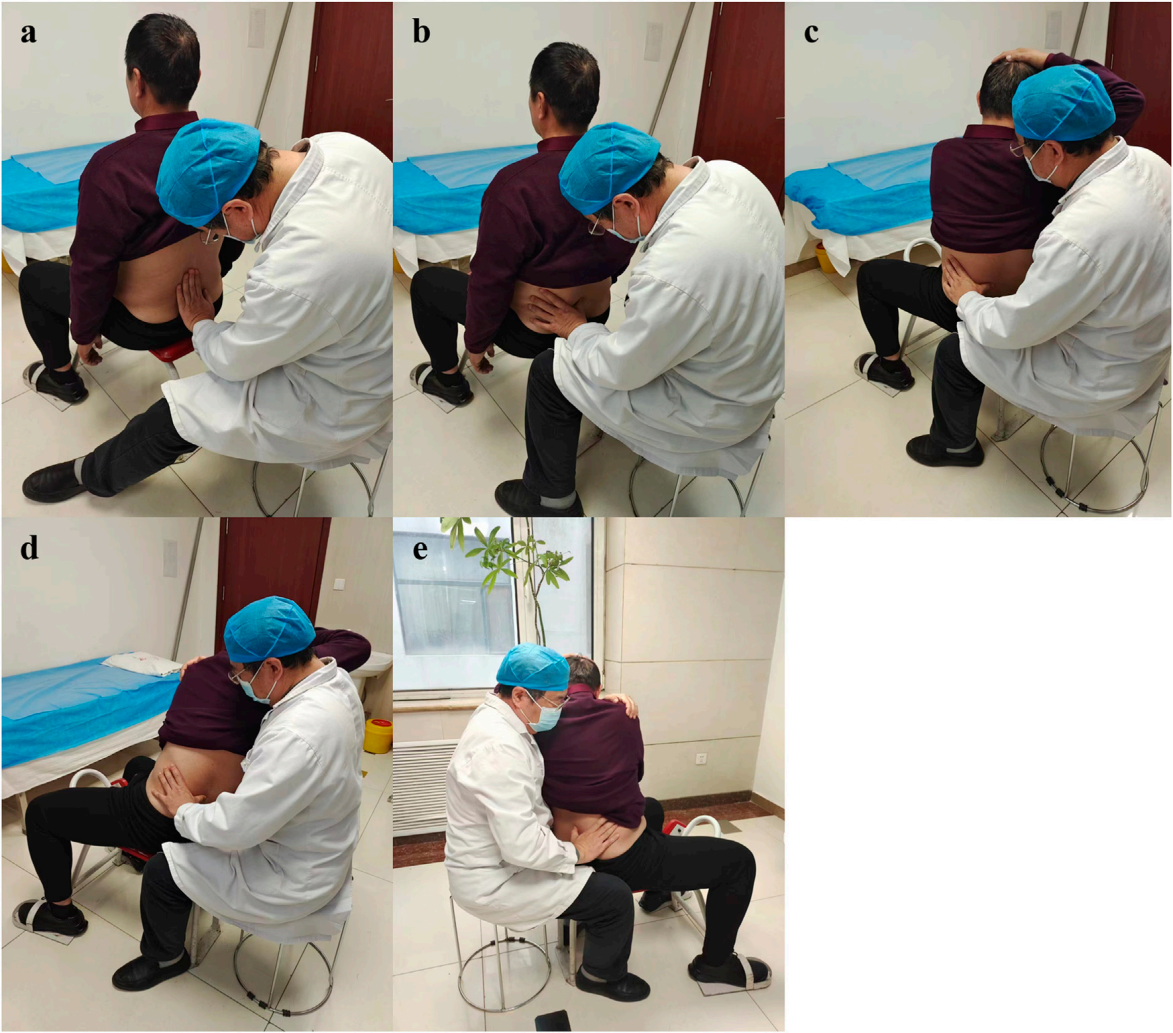
The operation procedure of lumbar fixed point rotational reduction manipulation. **(A)** The patient is seated in a chair; **(B)** The physician palpates with one thumb to determine the spinous process with the most pain; **(C)** The right hand of the patient is held behind the head, the left hand is wrapped around the waist to the right, and the doctor uses the right hand to reach forward through the right armpit of the patient and grasp the left shoulder of the patient, meanwhile, uses the left thumb tip to hold the tenderness point and guide the patient to bend forward in the lumbar spine; **(D)** The doctor uses his right hand to gently drive the patient’s left shoulder to the right and back, guide the patient to rotate the lumbar spine to the limit position, and then continue to rotate to the right while pulling upward; **(E)** The patient’s left and right hands were exchanged, rotated to the opposite side, and the above treatment was repeated.

Lumbar fixed point rotational reduction manipulation: 1. The patient is seated in a chair with his or her back to the physician, who palpates with one thumb to determine the spinous process with the most pain (The press position of each spinous process is shown in the Figure 1. Select the position where the tenderness is most obvious); 2. The right hand of the patient is held behind the head, the left hand is wrapped around the waist to the right, and the doctor uses the right hand to reach forward through the right armpit of the patient and grasp the left shoulder of the patient; 3. The doctor uses the left thumb tip to hold the tenderness point and guide the patient to bend forward in the lumbar spine. The doctor’s right shoulder pushes the patient’s right armpit to the left and upward, and tilts to the left to form a incline angle, so that the apex of the incline angle is located at the manual pressure point; 4. The doctor uses his right hand to gently drive the patient’s left shoulder to the right and back, guide the patient to rotate the lumbar spine to the limit position, and then continue to rotate to the right while pulling upward, at this time the doctor’s left thumb can touch the slight movement or accompanied by a flicking sound; 5. The patient’s left and right hands were exchanged, rotated to the opposite side, and the above treatment was repeated (Figure 2).

**FIGURE 3:**
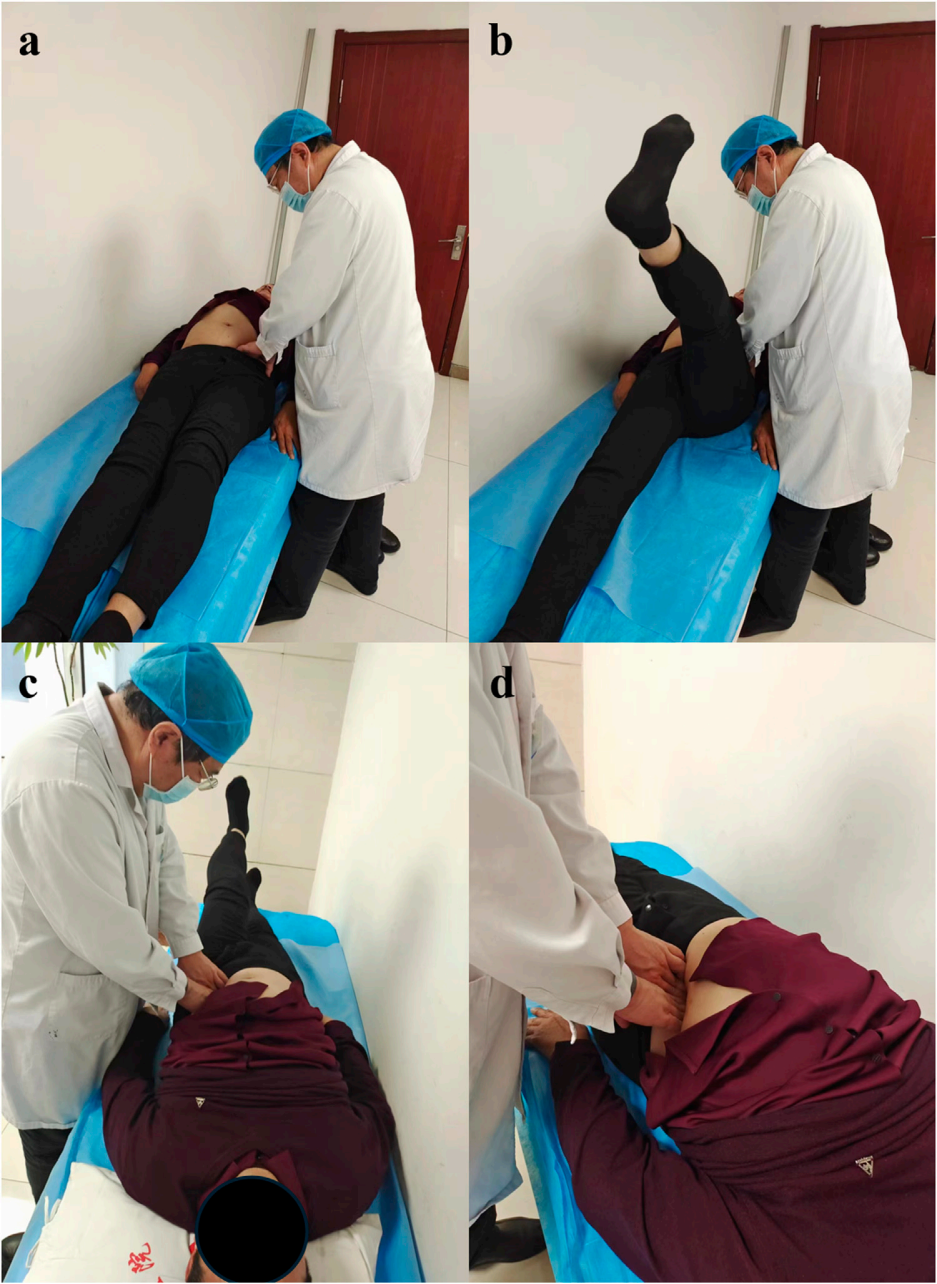
The operation procedure of abdominal release manipulation. **(A)** The patient was supine and asked to relax; **(B,C)** The doctor stood on the affected side of the patient, and palpated the abdomen of the fingers with four fingers together on the iliac fossa in the affected side Press down and gently knead the tender point. At the same time, ask the patient to raise the leg straightly on the affected side to the limit and then put it down; **(D)** The pressing position is shown in different perspective.

Abdominal release manipulation: 1. The patient was supine and asked to relax; 2. The doctor stood on the affected side of the patient, and palpated the abdomen of the fingers with four fingers together on the iliac fossa in the affected side (sliding palpation along the front of the iliac bone to the inner and lower depth) to find the tender point or the cord to determine the manipulative area; 3. Press down and gently knead the tender point. At the same time, ask the patient to raise the leg straightly on the affected side to the limit and then put it down. Repeat 10 times (Figure 3).

All patients were treated with the above two manipulations. Lumbar fixed point rotational reduction manipulation was difficult, and all patients were performed by the same senior orthopedic traditional Chinese medicine doctor (more than 30 years of experience). All the abdominal release manipulation were treated by traditional Chinese medicine doctors who had been practicing in orthopedics for more than 3 years. During the treatment, only the necessary communication with the patient is carried out, so as to reduce the experimental bias caused by mental and psychological factors.

### Data collection and radiographic measurement

Demographic information including age, sex, BMI were extracted from patient records. WOMAC scores were collected for all patients before and after treatment.

**FIGURE 4:**
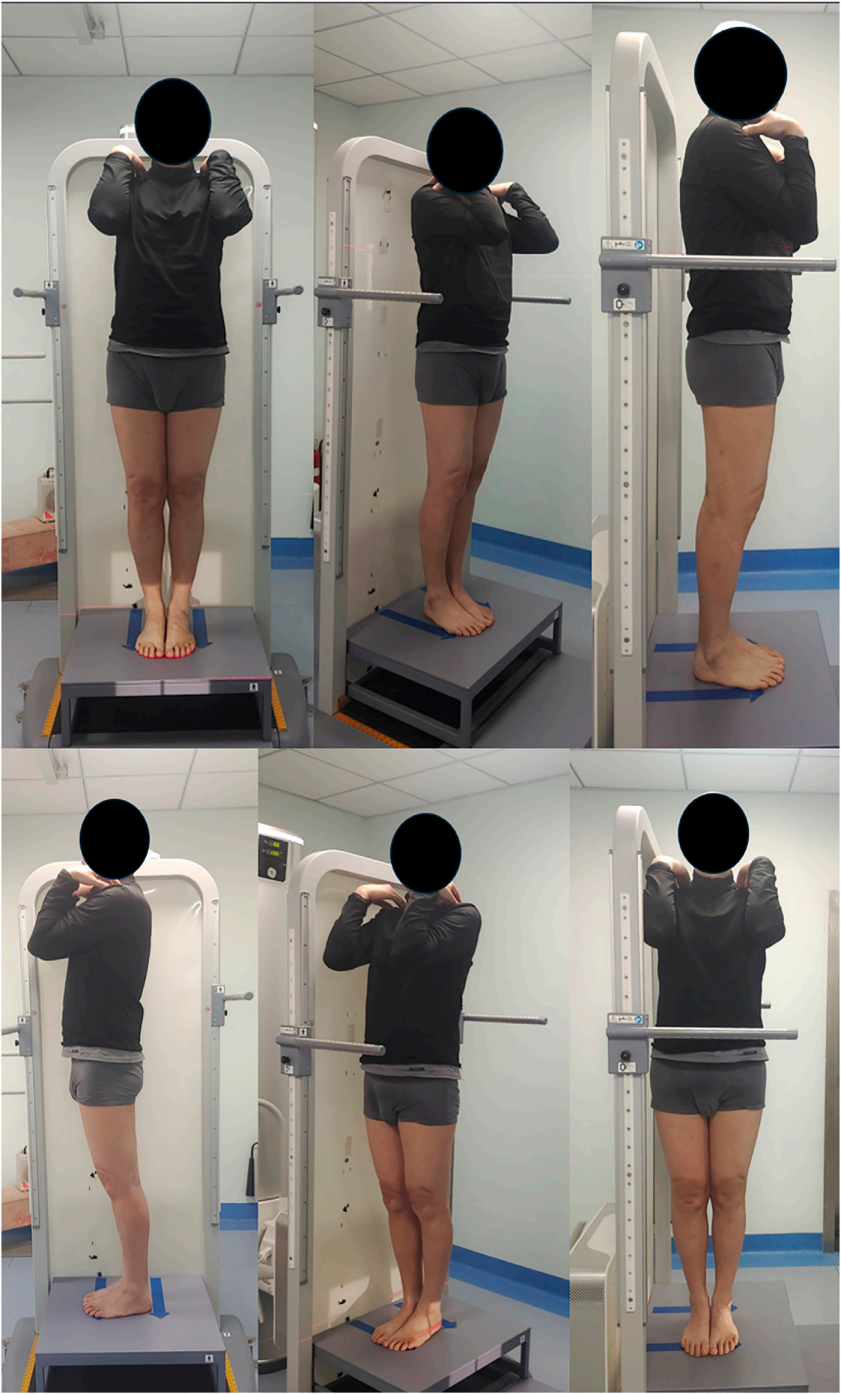
Full length lower limb X-ray in standard standing position in different perspective: The patient stood with his back against the camera bed and was upright facing the ball tube. Put his hands on the shoulders, and the inside of the calcaneus and the inside of the first metatarsal bone were attached to each other, the patella and toe were facing the front, and the back edge was close to the camera bed.

The method of X-ray of standard full weight bearing coronal position on both lower limbs and Coronal position of lumbar spine Using the Optima XR646 HD digital medical Radiography system. The inspection bed was adjusted to the standing vertical position, and the standing support plate was adjusted to the highest point in the movable position (about 35 cm from the ground). The distance between the focus of the bulb and the detector was 100 cm. The exposure range was as follows: the upper edge included L1 vertebra and the lower edge included ankle joint. The patient stood with his back against the camera bed and was upright facing the ball tube. Put his hands on the shoulders, and the inside of the calcaneus and the inside of the first metatarsal bone were attached to each other, the patella and toe were facing the front, and the back edge was close to the camera bed (Figure 4).

Using the panoramic photography method, the bulb was automatically exposed from top to bottom, while the detector moved with the bulb from top to bottom, and the continuous exposure computer automatically splicted the full-length weight-bearing position of both lower limbs and the X-ray images of the lumbar spine.

**FIGURE 5:**
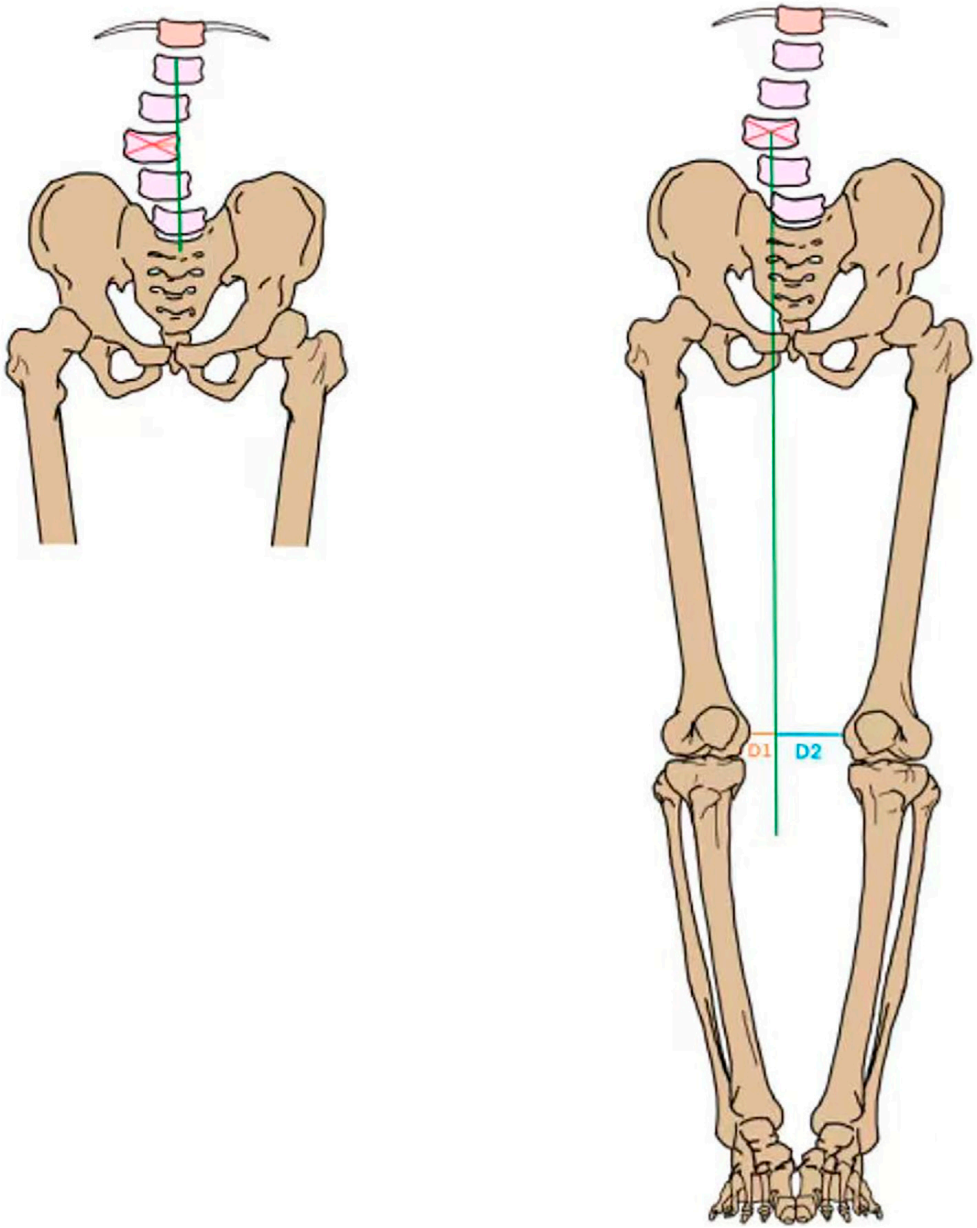
Take L3-SOD as an example (left). 1. Determine the center of the L3 vertebral body (red line); 2. Draw the Center Sacral Vertical Line (green line); 3. L3-SOD is the distance between the center of the vertebral body and CSVL (orange line). Take ΔL3-KOD for example, (right). 1. Determine the center of the L3 and make a line perpendicular to the ground (red line). 2. Measure the distance D1 and D2 (orange and blue lines) from the medial margin of both knees to this vertical line. 3. ΔL3-KOD is the absolute value of the difference between D1 and D2.

All images were measured using the MI platform software’s own ruler and angle measurement tool, recording the patient’s affected side and healthy side lateral patello-femoral angle (LPFA)\mechanical lateral distal femoral angle (mLDFA)\mechanical medial proximal tibial angle (mMPTA)\mechanical lateral distal tibial angle (mLDtA)\joint line convergence angle (JLCA)\hip-knee-ankle angle (HKA)\angle between mechanical axis and anatomical axis of femur (AMA)\mechanical shaft deflection (MAD)\coronal pelvic inclination (cPI), The angle between femoral anatomic axis and ground vertical line at coronal position was defined as the coronal femoral anatomic axis inclination (cFAI),the angle between the femoral mechanical axis and the ground vertical line was defined as the coronal femoral mechanical axis inclination (cFMI), similarly, the angle between the anatomical axis of the tibia and the ground vertical line was defined as the coronal tibial anatomic axis inclination (cTAI),the angle between the mechanical axis of the tibia and the ground vertical line was defined as the coronal femoral mechanical axis inclination (cTMI). The distance between the center of the lumbar vertebra and the vertical line of the sacrum was defined as the lumbosacral offset distance (L-SOD) (Figure 5), and the vertebra with the largest L1-L5 offset was defined as the vertebra with the most offset lumbosacral distance. The lumbosacral offset distance of the lumbar vertebra was defined as the maximum lumbosacrum offset distance (mL-SOD) of the patient. A vertical line perpendicular to the ground was established along the center of the lumbar vertebra, and the absolute value of the difference between the vertical line and the medial condylar margin of both sides of the femur was positioned as the lumbar knee offset distance (ΔL-KOD) of the vertebra, and the maximum ΔL-KOD corresponding to the L1-L5 vertebra was defined as the most offset lumbar knee distance from the vertebra (Figure 5). The lumbo-knee offset distance of this vertebra is defined as the patient’s maximum offset lumbo-knee distance (mΔL-KOD). Meanwhile, collected patients’ pelvic incidence (PI), lumbar lordosis (LL), pelvic tilt (PT), sacral slope (SS), the sagittal tibial anatomic axis inclination (sTI), femoropelvic angle (FPA), and femoral tilt angle (FTA) in sagittal position. All measurements were taken by three orthopedic surgeons at the same time, and the average of the three measurements was taken.

SPSS 22.0 (IBM, Armonk, NY, United State) statistical software was used for analysis, and *P* < 0.05 was considered statistically significant. The measurement data were compared between the two groups and analyzed by Paired Sample T-test or Wilcoxon rank testing according to whether the difference between the two pairs of matching data conforms to normal distribution. The measurement data between the two groups were compared using the independent sample T-test or Mann-Whitney test, according to the normal distribution and homogeneity of variance. *P*-value<0.05 was considered to be statistically significant. Continuous variables with normal distribution were presents as mean ± standard deviation (SD); non-normal variables were reported as median (interquartile range).

## Results

A total of 194 patients were enrolled in the study, 94 in the OMT group and 100 in the DT group. Other demographic parameters are shown in Table 1. There was no significant difference in demographic parameters between the two groups.

**TABLE 1:** Demographic parameters of patients in the OMT and DT groups.

	OMT group	DT group	
Age (years)	62.38 ± 8.38	61.70 ± 6.58	0.527
Sex (n (%))			0.165
Male	20 (21.28)	30 (30.00)	
Female	74 (78.72)	70 (70.00)	
Body mass index (kg/m^2^)	25.68 ± 3.04	24.96 ± 2.86	0.090
K-L Grade (n (%))			0.362
Ⅰ	2 (2.13)	5 (5.00)	
Ⅱ	42 (44.68)	53 (53.00)	
Ⅲ	34 (36.17)	30 (30.00)	
Ⅳ	16 (17.02)	12 (12.00)	

Table 2 shows the WOMAC scores of the OMT and DT groups before and after intervention and the comparison between the two groups. It can be seen that there was no significant difference in WOMAC scores between the two groups before treatment, and after intervention treatment, WOMAC scores in both groups were significantly improved. However, there were significant differences in WOMAC scores between the OMT group and the DT group after treatment. The percentage of patients with pain decreased from 9 (7–11) before intervention to 5 (3–6.25) in the OMT group and from 8 (6–11) to 6 (4–7) in the DT group, with significant difference between the two groups after intervention (*P* = 0.025). In terms of function, after intervention, the two groups were 18 (12–22) and 26 (19–31), respectively, with significant differences (*P* < 0.001). After treatment, the final WOMAC total scores were 24 (17–31) and 33 (25–39), aslo respectively with significant differences (*P* < 0.001).

**TABLE 2:** WOMAC scores of OMT group and DT group before and after intervention.

	OMT group			DT group			*P*-value between OMT and DT group before treatment	*P*-value between OMT and DT group after treatment
	Before OMT	After OMT	P	Before DT	After DT	P		
WOMAC pain score	9 (7–11)	5 (3–6.25)	<0.001	8 (6–11)	6 (4–7)	<0.001	0.191	0.025
WOMAC stiffness score	4 (2–5)	2 (1–3)	<0.001	3 (2–4)	2 (1.25–3)	<0.001	0.121	0.082
WOMAC function score	30 (24–35.25)	18 (12–22)	<0.001	33 (23–36)	26 (19–31)	<0.001	0.338	<0.001
WOMAC total score	43 (35–49)	24 (17–31)	<0.001	42 (35.25–49)	33 (25–39)	<0.001	0.998	<0.001

The force lines of the lower limbs on the affected side and healthy side of the patient are shown in Table 3. It can be seen that the global force lines of lower limbs (LPFA, mLDFA, mMPTA, mLDTA, JLCA, HKA, AMA, MAD) on both the affected and healthy sides did not change significantly before and after treatment. cFMI, cFAI, and cTAI on the affected side of the local force line not changed significantly, and only cTMI increased significantly (*P* = 0.034), while the local force line on the healthy side did not change significantly.

**TABLE 3:** Changes of lower limb force line before and after OMT.

	Affected side			Healthy side		
	Before OMT	After OMT	P	Before OMT	After OMT	P
LPFA	94.19 ± 5.82	94.19 ± 5.49	0.943	94.55 ± 6.29	94.75 ± 5.75	0.767
mLDFA	87.83 ± 2.65	87.73 ± 2.66	0.262	87.81 ± 2.69	87.63 ± 2.69	0.301
mMPTA	86.45 (84.30–87.90)	86.20 (84.375–87.90)	0.725	86.42 ± 2.55	86.66 ± 3.00	0.149
mLDTA	90.48 ± 2.69	90.55 ± 2.91	0.527	90.67 ± 3.06	90.62 ± 2.86	0.871
JLCA	2.35 (1.375–3.40)	2.25 (1.50–3.50)	0.454	2.35 (1.575–3.125)	2.40 (1.50–3.40)	0.164
HKA	176.90 (173.80–179.525)	176.60 (172.975–179.40)	0.140	177.05 (174.95–179.30)	177.05 (174.90–179.35)	0.719
AMA	6.92 ± 1.06	6.91 ± 1.12	0.887	6.89 ± 1.03	6.94 ± 1.06	0.266
MAD	8.00 (0.00–18.00)	8.50 (1.00–20.00)	0.557	7.00 (1.00–16.00)	7.50 (0.75–14.25)	0.266
cFMI	3 (1.375–4.25)	3.2 (1.575–4.20)	0.178	3.18 ± 1.80	3.13 ± 1.85	0.568
cTMI	6.56 ± 2.93	6.79 ± 2.40	0.034	6.25 (4.90–7.725)	6.2 (5.00–7.40)	0.686
cFAI	8.75 (5.75–10.95)	8.45 (6.075–10.30)	0.695	9.40 (7.575 10.60)	9.20 (7.575–10.625)	0.442
cTAI	5.90 (4.70–7.80)	6.35 (4.775–8.125)	0.559	5.95 (4.225–7.625)	6.05 (4.20–7.35)	0.712

LPFA, lateral patello-femoral angle; mLDFA, mechanical lateral distal femoral angle; mMPTA:mechanical medial proximal tibial angle; mLDTA, mechanical lateral distal tibial angle; JLCA, joint line convergence angle; HKA, hip-knee-ankle angle; AMA, angle between mechanical axis and anatomical axis of femur; MAD, mechanical shaft deflection; cFAI, coronal femoral anatomic axis inclination; cFMI, coronal femoral mechanical axis inclination; cTAI, coronal tibial anatomic axis inclination; cTMI, coronal femoral mechanical axis inclination.

For angles that are normally distributed, we use Mean ± SD, to describe them. For angles that are not normally distributed, we use the description P50 (P25-P75).

Table 4 represents the changes in pelvic and spinal parameters in the coronal position. It can be seen that mL-SOD changed significantly before and after treatment, from 9.01 ± 6.61 cm before OMT to 7.53 ± 6.34 (*P* < 0.001). Similarly, mΔL-KOD also decreased significantly after treatment. It changed from 27.15 ± 18.34 to 22.72 ± 18.13 (*P* = 0.001). The pelvic tilt angle did not change significantly before and after treatment.

**TABLE 4:** Changes of coronal spinal-pelvic parameters before and after OMT.

	Before OMT	After OMT	P
mL-SOD	9.01 ± 6.61	7.53 ± 6.34	<0.001
mΔL-KOD	27.15 ± 18.34	22.72 ± 18.13	0.001
cPI	1.6 (0.80–2.60)	1.4 (0.90–2.425)	0.944

cPI, coronal pelvic inclination (cPI); mΔL-KOD, maximum lumbar-knee offset distance; mL-SOD, maximum lumbosacrum offset distance.

Table 5 represents the changes in sagittal position parameters of patients before and after treatment. It can be seen that LL of patients increased significantly after treatment (*P* = 0.036). There were no significant changes in PI(*P* = 0.569), PT (*p* = 0.671), SS(*P* = 0.137). FTA in sagittal position decreased significantly (*P* = 0.026), while sTI(*P* = 0.202) and FPA (*p* = 0.726) did not change significantly.

**TABLE 5:** Changes of sagittal position parameter before and after OMT.

	Before OMT	After OMT	P
LL	30.37 ± 12.07	31.78 ± 12.57	0.036
PI	51.85 ± 11.84	52.72 ± 11.37	0.569
PT	15.75 (9.35–23.325)	15.30 (7.175 ± 20.30)	0.671
SS	36.40 ± 10.02	37.74 ± 10.19	0.137
FTA	4.85 (2.7225–8.825)	4.75 (2.70–7.65)	0.026
sTI	6.45 (3.625–9.45)	6.40 (4.475–9.95)	0.202
FPA	9.33 ± 14.30	9.48 ± 12.83	0.726

PI, pelvic incidence; LL, lumbar lordosis; PT, pelvic tilt; SS, sacral slope.

sTI, the sagittal tibial anatomic axis inclination; FPA, femoropelvic angle; FTA, femoral tilt angle.

## Discussion

KOA, a chronic joint disease involving the whole knee joint, is the most common joint disease worldwide, with KOA changes observed on X-rays in approximately 61% of adults aged older than 45 years (Nelson et al., 2021). OMT, as a kind of physical therapy, has been proved effective in relieving pain and improving knee function in both short (Pozsgai et al., 2022) and long term (Nigam et al., 2021), but the mechanism is unclear. In our study, OMT showed significant short-term (2 weeks) improvements in both pain, stiffness, and knee function, and the effect was also significant due to the DT Group. The OMT involved in this study has not been seen in previous literature, so it cannot be compared with previous studies. For more strictly explain the mechanism of the two manipulations involved in this study, we asked the patient to take full-length X-ray of the lower extremity in standard posture before and 2 weeks after treatment, which is the first time that the X-ray posture was proposed, so as to better exclude the bias caused by the shooting posture and more accurately measure the subtle changes in the lower extremity force line caused by the manipulative treatment.

There are many reasons for knee pain and functional limitations in patients with KOA, which are mainly divided into two aspects: 1. Inflammation caused by intra-articular tissue injury and degeneration (Liu-Bryan and Terkeltaub, 2015); 2. Changes in mechanical stress within the joint (Eskelinen et al., 2020). Non-steroidal anti-inflammatory drugs (NSAIDs) are generally the first choice for anti-inflammatory therapy for inflammatory response. However, Adatia et al. (2012) studies suggest that inflammatory response is not the key cause of pain, but the source of pain likely stems from the richly innervated synovium, subchondral bone and periosteum components of the joint. A study by Bandak et al. (2021) also showed that KOA patients who maintained exercise had no change in knee inflammatory activity, but decreased pain. And pain is thought to be the main factor that leads to functional changes (Stauffer et al., 1977). This both suggests that changes in mechanical stress in the joints may be the main source of symptoms in patients with KOA. This study also reached a similar conclusion, after treatment, both patients in the DT and OMT groups had significant improvement in symptoms. Improvements in all components of the WOMAC score in the DT group were similar to previous study (Tannenbaum et al., 2004). On average, there was no significant difference in WOMAC between the two groups before treatment, but OMT treatment was superior to drug therapy in improving pain and function after treatment.

**FIGURE 6:**
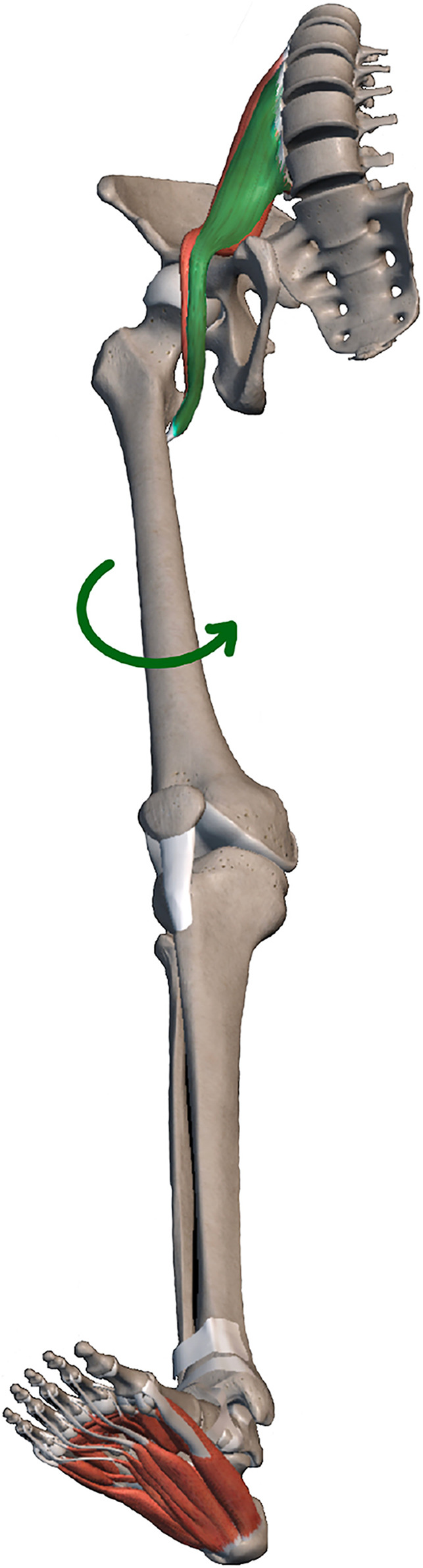
After OMT intervention, the iliopsoas muscle changed from tense (green) to relaxed (red), and the femur rotated.

There are two types of OMT used in this study: 1. Lumbar fixed point rotational reduction manipulation (a kind of osteopathic manipulation) is a method to adjust patients’ facet joint syndrome through changes in their own posture and external traction, so as to change the muscle tension of the lumbar muscles (Cohen and Raja, 2007). 2. Abdominal release manipulation: Release superficial abdominal muscles such as external oblique, internal oblique, transverse abdominis, rectus abdominis, and deep abdominal muscles such as psoas major and quadrate psoas. Thus improve the spine and pelvic force line, indirectly correct the lower limb force line. After OMT treatment, there was no significant change in the overall line of force of the lower limbs in the coronal position. However, the local force line in the coronal position was slightly changed, and the cTMI became larger. Interestingly, cTAI, cFMI and cFAI did not change. In a study of 14 fresh frozen cadavers, Alves-da-Silva et al. (2019) showed that the tibia and fibula rotated according to the motion of the knee joint. In our manual treatment, abdominal release manipulation intervene the psoas major muscle, improved muscle function and reduced muscle tone (Matsuda et al., 2021),which may cause slight internal rotation of the femur (Siccardi et al., 2015). The tibia, like a magnifying glass, is accompanied by a more obvious rotation of the femur, which is also the reason why cTAI is not changed but the cTMI is changed——the tibia has a rotation centered on its anatomical axis. This slight rotation is not well represented on previous lower limb X-rays because the mechanical axis and anatomical axis of the tibia are parallel in the natural standing position, but in this study, the standard standing posture with feet together was used to reflect this slight difference (Figure 6).

Since Rahbar et al. (2015) reported the relationship between hip, knee and waist in 2015, the spine-pelvi-lower limb force line has gradually become a research hotspot. Segi et al. (2023) demonstrated that an imbalance in the spine pelvis can lead to a shift in center of gravity, which have a significant impact on knee pain and function (Naili et al., 2018). In this study, mL-SOD and mΔL-KOD were measured, representing shifts in the trunk’s center of gravity relative to the pelvis and relative to the knee joint, respectively. After OMT treatment, the mL-SOD was significantly improved, but the pelvis itself did not change significantly in the coronal plane, demonstrated that OMT can correct the lumbar spine sequence through the treatment of lumbar muscles and facet joints, thereby reducing knee stress stimulation, relieving knee pain and improving knee function.

Previous studies have focused on the spinal, pelvi-lower limb force line in sagittal position. Lee et al. (2013) proposed that FPA is a better parameter to describe pelvic compensation than PT, and FTA can represent the degree of knee flexion, the increase of FTA will lead to the straightening of lumbar curvature. After OMT treatment, patients LL became larger and FTA became smaller. This means that the patient’s knee flexion is reduced and the lumbar curvature is restored. On this basis, there were no significant changes in the patient’s pelvis. Lee et al. (2013) proposed knee flexion compensatory mechanism states that the lumbar spine compensates first when the lumbar spine is highly mobile, while the pelvis compensates through rotation and hip flexion when the lumbar spine is stiff. In this study, OMT was mainly used for lumbar muscle intervention, so we believe that LL changes are the initiating factor, and minor lumbar curvature changes will be compensated by the knee joints first. In summary, OMT can relieve the lumbar muscle, improve the small joint disorder, improve the lumbar offset in the coronal position, and increase the lumbar curvature in the sagittal position. There is also rotation of the femur and straightening of the knee in the sagittal position, both of which can improve the mechanical stress stimulation of the knee and thus reduce symptoms in patients with KOA.

This study, as the first to propose full-length X-ray of the lower limbs in a standard posture and the first to show the effect of OMT on imaging, has the following shortcomings: 1. Because OMT techniques have different effects from different physicians, the same physician was used in this study to treat patients with lumbar fixed point rotational reduction manipulation, which may also explain the low popularity of OMT and may lead to certain bias. 2. In consideration of ethical requirements to avoid patients being exposed to excessive unnecessary radiation, we did not conduct imaging examinations before and after treatment for patients in the DT group. 3. This study lacks long-term follow-up, and the comparison of long-term efficacy of OMT and DT is unclear. 4. Due to the sample size, this study could not match the propensity score of patients in the OMT group and the DT group. 5. This study did not distinguish between genu varus and genu varus deformity, and different malformations may correspond to different changes in muscle tone. 6. This study is a retrospective cohort study, and further RCT studies with a larger sample size are needed.

## Conclusion

Compared with drug therapy, osteopathic manipulative treatment can better improve WOMAC scores in KOA patients. It relives symptoms by loosening muscles and correcting small joint disorders to improve local knee alignment.

## Data Availability

The original contributions presented in the study are included in the article/supplementary material, further inquiries can be directed to the corresponding authors.
